# Sex and Age Affect Progression to Total Knee Arthroplasty After Cartilage Surgery: A UK Biobank Cohort Study

**DOI:** 10.1177/19476035261450100

**Published:** 2026-06-29

**Authors:** Nicola J. Kuiper, Charlotte H. Hulme, Martyn Snow, Martin Frisher, Karina T. Wright, Jan Herman Kuiper

**Affiliations:** 1 Oswestry/Keele Orthopaedic Research Group, The Robert Jones & Agnes Hunt Orthopaedic Hospital NHS Foundation Trust, UK; 2 Centre for Science and Technology in Medicine, School of Life Sciences, Keele University, UK; 3 The Royal Orthopaedic Hospital, Birmingham, UK; 4 School of Allied Health Professions & Pharmacy, Keele University, UK

**Keywords:** articular cartilage, cartilage lesions, total knee arthroplasty, sex differences, microfracture, debridement, unicompartmental arthroplasty, cartilage repair

## Abstract

**Objective:**

Evaluate the association of sex and age at surgery with time to total knee arthroplasty (TKA) following knee cartilage surgery in UK Biobank (UKB) participants.

**Design:**

Participants with a history of knee cartilage surgery were classified into four surgical subgroups: cartilage repair, debridement, microfracture, and unicompartmental knee arthroplasty. Time from index surgery to primary TKA in each group was analysed using the Kaplan-Meier method and the sexes compared using log-rank tests. Cox proportional hazards regression evaluated associations between age at surgery, sex, and surgery type with risk of progression to TKA.

**Results:**

The study included 1,967 males and 1,741 females. Log‑rank testing demonstrated significant sex‑based differences in time to TKA following debridement (*P* = 0.002) and microfracture (*P* = 0.003), with shorter TKA‑free survival observed in females. Cox regression showed that female sex was associated with a higher hazard of progression to TKA compared with male sex (hazard ratio [HR] = 1.19, *P* = 0.021). Increasing age at surgery was associated with increased risk (HR = 1.05 per year, *P* < 0.0001).

**Conclusion:**

Female sex and older age at surgery were associated with a higher risk of progression to TKA following cartilage surgery. Sex‑specific differences in time to TKA were most evident after debridement and microfracture. These findings describe associations observed within the constraints of available UKB data.

## Introduction

Adult cartilage has a limited capacity for self-repair and cartilage damage following an injury is a major cause of further cartilage loss and subsequent osteoarthritis.^1,2^ Articular cartilage lesions, whether focal or diffuse, disrupt the biomechanical integrity of the joint and pose a significant clinical challenge. The knee is especially susceptible to cartilage damage due to its complex biomechanics and weight-bearing function. A variety of surgical procedures are available for the repair of articular cartilage lesions (**Table 1**), each associated with variable clinical outcomes. The outcomes are influenced by patient-specific factors, including demographic characteristics such as sex and age at surgery, the nature and extent of the cartilage defect, and any prior surgical interventions.^3-5^ Consequently, selecting the most appropriate surgical procedure requires careful consideration of individual patient characteristics as well as the anticipated long-term success of joint preservation.

**Table 1.** Common Surgical Procedures to Manage Articular Cartilage Lesions Within the Knee.

Procedure	Surgical approach	Description and indication
Lavage^6^	Arthroscopic	Minimally invasive irrigation of the joint space to remove debris; typically allows for immediate postoperative weight-bearing.
Debridement / Chondroplasty^7,8^	Arthroscopic	Mechanical smoothing of damaged cartilage and removal of loose fragments to address mechanical symptoms.
Microfracture^9,10^	Arthroscopic	Creation of channels into the subchondral bone at the lesion site (typically <2.5 cm^2^) to facilitate cell migration and infiltration of growth factors.
Autologous matrix-induced chondrogenesis (AMIC)^11^	Arthroscopic or Mini-open	A single-step procedure combining marrow stimulation with the application of a collagen membrane to stabilise the clot.
Autologous chondrocyte implantation (ACI)^12,13^	Two-stage procedure	Harvest of patient chondrocytes for *in vitro* expansion, followed by re-implantation into the lesion site under a membrane.
Mosaicplasty^14^	Arthroscopic or Arthrotomy	Relocation of cylindrical osteochondral plugs from low-load donor areas to the focal defect site.
Unicompartmental joint arthroplasty^15,16^	Open surgery	Resurfacing of a single degenerate compartment of the joint, preserving the healthy compartments and cruciate ligaments.
Osteotomy^17^	Open surgery	Surgical realignment of the joint axis to redistribute mechanical load away from the area of the cartilage lesion.

Sex-based differences in knee cartilage injury and repair outcomes are increasingly recognised as clinically significant.^18^ A recent systematic review and meta-analysis by Fattini Fellini *et al*.^19^ assessed whether sex influences outcomes following intra-articular treatments for knee osteoarthritis, including corticosteroids, platelet-rich plasma, hyaluronic acid, and cell-based therapies. Across 848 studies including at least 99,000 patients, only 2.5% of studies reported sex-stratified analyses, highlighting a major gap in the literature. Among the paucity of data, no statistically significant differences were observed between men and women in relation to pain relief or functional improvement, but the wide confidence intervals (CI) suggested the available data was probably underpowered to detect clinically relevant differences. The authors emphasised the importance of sex-specific analyses to clarify potential biological or hormonal influences on long-term outcomes. Beyond patient-reported outcomes, other evidence suggests clinically relevant sex differences following knee cartilage injury and surgery. Women frequently report higher post-injury and postoperative pain levels than in men,^20^ and may experience a more rapid progression to osteoarthritis following cartilage damage.^21^ Further, women tend to have smaller cartilage volume and thickness in their knee compared with men, even after adjusting for body size and height.^22^ A systematic review and meta-analysis of 1,468 athletes across 22 primary studies reported that female athletes may face greater challenges in returning to sport following contemporary cartilage procedures in the knee, such as autologous chondrocyte implantation (ACI) or mosaicplasty when compared with male athletes.^23^ Data from the German Cartilage Registry indicate that women undergoing cartilage surgery tend to be older, have longer symptom duration, and report lower baseline KOOS (Knee injury and Osteoarthritis Outcome Score) compared with men.^24^ These differences are thought to arise from a combination of biomechanical, hormonal, and molecular factors. Collectively, these findings suggest that both sex and age at surgery may influence long-term cartilage repair outcomes.

While patient‑reported outcome measures and imaging assessments provide valuable insight into short‑ and mid‑term recovery following cartilage repair, time to total knee arthroplasty (TKA) represents a definitive and clinically meaningful endpoint reflecting failure of joint preservation. Assessment of survival time to TKA enables evaluation of the long‑term effectiveness of cartilage surgery in delaying or preventing progression to end‑stage joint disease. Although established registries such as the German Cartilage Registry are designed to capture longitudinal outcomes, including indicators of osteoarthritis progression, they do not currently record conversion to TKA. More broadly, many existing cartilage registries lack routine capture of TKA as an outcome, thereby limiting the ability to investigate long‑term joint survival following cartilage procedures.

The UK Biobank (UKB) is a large-scale, prospective cohort study that enrolled approximately 500,000 participants aged 40 to 69 years from across the United Kingdom between 2006 and 2010.^25,26^ Its primary objective is to facilitate research into the genetic and environmental determinants of human diseases. UKB provides a unique opportunity to examine long-term outcomes following knee cartilage surgery, including progression to TKA, in a large, population-based cohort. The aim of this study was to evaluate the association of sex and age at surgery with time to TKA following knee cartilage surgery among UKB participants.

## Methods

### Source of Study Participants

This observational study was reported in accordance with the STROBE guidelines, and the completed checklist is provided as Appendix 1 in the Supplementary Material. At their recruitment, all UKB participants gave informed consent to participate and to be followed up. All procedures involving UKB data were conducted in accordance with the ethical standards of the UKB Ethics and Governance Framework. Ethical approval for UKB was obtained from the Northwest Multi-centre Research Ethics Committee, UK (REC reference: 11/NW/0382). Permission to access and analyse UKB data was approved under UKB application study number 280752. Mortality data for censoring were obtained through national death registries linked to UKB records. Surgical treatment records and mortality data were extracted from the UKB between May and July 2025. To ensure ethical compliance and data integrity, the cohort was screened against the latest UKB withdrawal logs in December 2025, and any participants who had rescinded consent were removed prior to analysis.

### Study Participants With a History of Knee Surgery

All data manipulation was conducted using RStudio Posit Workbench on the UKB Research Analysis Platform (UKB-RAP). Surgical procedure data were derived from the UKB dataset using the following strategy. From the outset, participants who underwent knee surgery were identified within the UKB dataset using OPCS-4 procedure codes (Office of Population Censuses and Surveys Classification of Surgical Operations and Procedures, 4th revision) and by reviewing the summary of operations data.^27^ The OPCS code lists for knee operations – manually curated in collaboration with the Clinical Coding Team at the RJAH Hospital – are shown in Appendix 2 in Supplementary Material. This process integrated OPCS knee operation codes with right/left sided laterality and anatomical compartmentalisation of the knee joint. Next, for each participant, the OPCS-4 codes were tabulated alongside participant birth year, sex, and mortality date.

OPCS codes are available for UKB participants via the incorporation of Hospital Episode Statistics (HES). Although HES were introduced in 1987, HES Admitted Patient Care data have only been available from NHS Digital UK since April 1998.^28^ Therefore UKB identified participants who had undergone knee surgery previous to 1998 using self-reported questionnaires at baseline interviews. A search of the questionnaires reported by the tabulated participants for instances of the term “knee” identified participants who had undergone previous knee surgery. There was no record of laterality of their previous knee surgery. Since the year of their previous knee surgery was not reported consistently, we used a binary classification; participants with complete data confirming the event were coded as “yes previous” while those where the event was not observed or those with missing or insufficient data were coded as “no previous.”

### Surgical Subgroups and Procedure Date Order

Study participants with HES-recorded knee surgery were sorted into surgical subgroups based on the type of articular cartilage repair procedure. Study participants who underwent ACI, AMIC, and/or Mosaicplasty were pooled due to limited sample sizes within these procedure categories. The following surgical subgroups were established: “Cartilage repair” (including ACI, AMIC, Mosaicplasty), “Debridement” (including Debridement and Chondroplasty), “Microfracture” and “Unicompartmental.” Some participants underwent more than one type of cartilage repair procedure and were therefore included in multiple cartilage repair subgroups.

Next, the incidence and date of a primary TKA among the study participants was recorded. The final steps involved matching the surgical procedure dates to each surgical subgroup and then putting these procedure dates into their chronological order. The interval between two surgical procedures (e.g. between cartilage procedure and TKA) and the participant’s age at the time of intervention were calculated to facilitate the evaluation of long-term outcomes. The derived tabulated UKB dataset included participant birth year, gender, sex, date of death if applicable, laterality of surgical procedure, surgical subgroups, procedure date order, interval between procedures, participant’s age at intervention, incidence, and date of primary TKA.

### Statistical Analyses

All analyses were performed using RStudio Posit Workbench (R version 4.4.0) on the UKB-RAP with the R packages tidyverse, survival and ClinicalTrialSummary.^29-31^ Continuous variables were presented as mean (range) or mean (SD), depending on data distribution. For the survival analysis, failure was defined as conversion to TKA. Time‑to‑event was calculated from the date of the cartilage surgical procedure to the earliest of: (a) the date of TKA or (b) the last date of follow‑up without TKA. Participants who died during follow‑up were censored at the date of death, as death does not constitute failure of the index procedure but represents a competing terminal event. Kaplan-Meier survival curves were generated to estimate survival probabilities, and group comparisons were assessed using the ratio of restricted mean times lost (RRMTL) and the log‑rank test (*P* < 0.05 considered statistically significant).^31^ The RRMTL is a summary measure comparable with the hazard ratio (HR) but is also valid when the proportional hazard (PH) assumption does not hold. When the PH assumption holds approximately, its value is close to the HR.

Cox proportional hazards regression was used to evaluate the association between covariates and risk of progression to TKA. The model included sex, age at surgery, and procedure type, with an interaction term between sex and procedure type. To assess if the proportional hazard assumption held approximately, Kaplan-Meier survival curves were compared between the strata (sex, procedure), and scaled Schoenfeld residual plots by time for age were checked to see if they were approximately horizontal. HRs and 95% CI were reported relative to reference groups.

## Results

### Demographics of the Surgical Subgroups From the UKB

A total of 3,708 study participants (1,967 Male, 1,741 Female) were identified. The demographics for each surgical subgroup and their survival interval to TKA are shown in **Table 2**. The age distributions for males and females in each surgical subgroup were comparable. This is consistent with the narrow age range used for recruitment to the UKB. The UKB study population was defined as individuals aged 40 to 69 years at the time of recruitment (2006-2010).

**Table 2.** Demographics of Surgical Subgroups Derived From the UK Biobank.

Procedure	Sex	N	Age (years) at index surgery median (IQR) + range	Interval to TKA (Years) Mean (SD)
Cartilage repair	Male	28	55 (45-50) 37-74	15 (7)
(*n* = 64)	Female	36	54 (43-59) 34-81	13 (7)
Debridement	Male	1517	59 (53-65) 35-82	11 (6)
(*n* = 3251)	Female	1734	60 (54-66) 35-82	10 (6)
Microfracture	Male	190	55 (50-63) 40-74	11 (4)
(*n* = 382)	Female	192	56 (51-63) 40-74	11 (5)
Unicompartmental	Male	199	63 (57-69) 45-77	10 (4)
(*n* = 409)	Female	210	63 (57-70) 46-82	10 (4)

IQR, Interquartile Range; SD, Standard Deviation; TKA, Total Knee Arthroplasty.

### Cartilage Knee Surgery Pathways Based on Their Chronological Order

**Figure 1.**
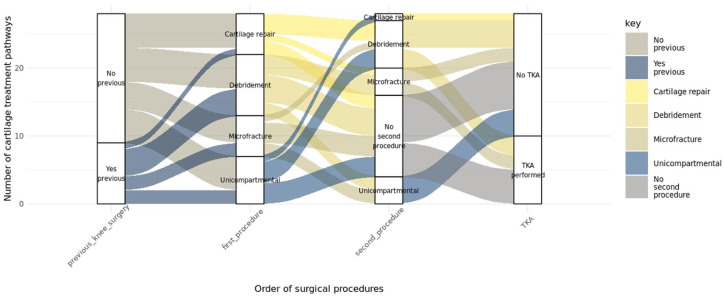
Alluvial plot to illustrate all possible journeys of study participants who had articular cartilage treatment. The x axis represents sequential stages in chronological order: previous knee surgery, first cartilage procedure, second cartilage procedure, and TKA. Each column represents the surgical categories at each stage, including a category for participants who did not undergo a second procedure. The journeys of participants are represented by coloured bands that flow through the stages and surgical categories. The key reflects the coloured bands. The y-axis shows the number of different cartilage treatment pathways. The width of each band represents the proportional number of participants following each trajectory.

An alluvial plot (**Fig. 1**), generated using the ggalluvial package, illustrates the 28 treatment trajectories observed among the 3,708 participants with cartilage‑related knee surgery.^32^ At baseline, 135 participants reported previous knee surgery (34 females, 101 males). Of these, 13 participants (3 females, 10 males) underwent two procedures, and 25 participants (7 females, 18 males) subsequently required a TKA. Most participants (90.1%) had undergone a single cartilage procedure, whilst 9.9% had two or more procedures before TKA. Debridement was the most common first procedure. Most participants who underwent unicompartmental arthroplasty first did not require TKA (218/252, 87%). A small number of participants transitioned between procedure types – for example, some patients moved from microfracture to unicompartmental arthroplasty (15/382, 3.9%). Only three participants had a third procedure, but these were too few to display in the alluvial plot. Overall, 773 of the 3,708 participants underwent a TKA (female 452, male 321).

### Survival Time to TKA for All Surgical Subgroups

**Figure 2.**
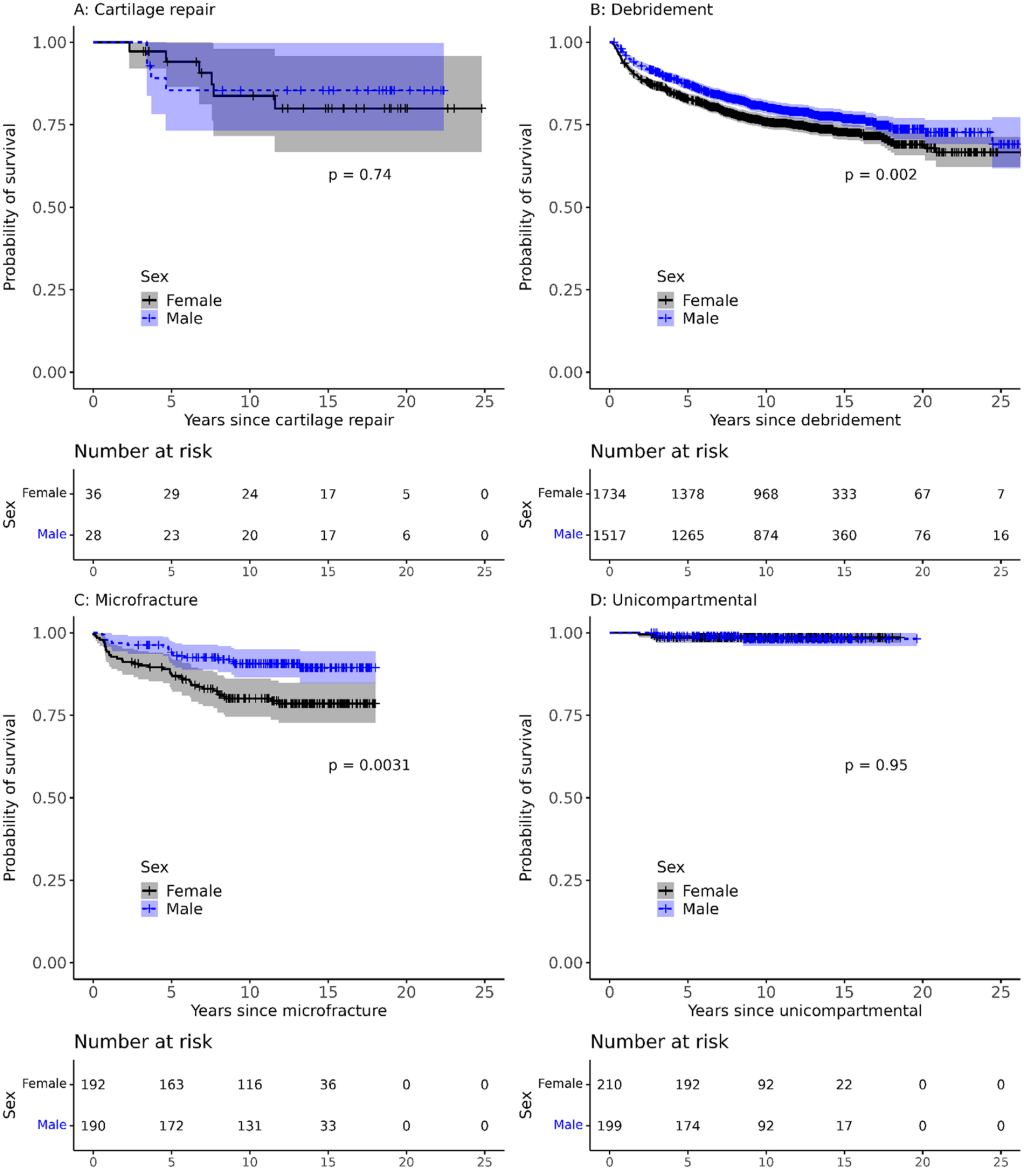
Twenty‑five-year survival curves to TKA for participants undergoing (**A**) Cartilage repair, (**B**) Debridement, (**C**) Microfracture, or (**D**) Unicompartmental knee arthroplasty. Kaplan-Meier survival estimates are shown with 95% confidence intervals. Numbers at risk beneath each curve indicate the number of participants remaining under follow‑up (event‑free and uncensored) at each time point, reflecting the available length of follow‑up over time. Participants who died during follow‑up were censored at the time of death (cartilage repair: *n* = 2; debridement: *n* = 184; microfracture: *n* = 9; unicompartmental arthroplasty: *n* = 10).

**Figure 2** shows the 25-year Kaplan-Meier survival curves for the risk of conversion to TKA across subgroups. The curves suggest that females undergoing debridement had an almost 20% higher risk of conversion to TKA (RRMTL 1.17, 95% CI 1.02-1.34, log-rank test *P* = 0.002) and those undergoing microfracture had more than double the risk (RRMTL 2.16, 95% CI 1.31-3.57, *P* = 0.003; **Fig. 2B and C**). No evidence for sex differences was observed for cartilage repair procedures (RRMTL 1.24, 95% CI 0.39-3.93, *P* = 0.74) or unicompartmental knee arthroplasty (RRMTL 0.99, 95% CI 0.47-2.11, *P* = 0.95; **Fig. 2A and D**).

Cox regression was used to examine the association of age and sex with progression to TKA (**Table 3**). Diagnostic plots suggested no meaningful deviation from the proportional hazard assumption, other than for sex within the cartilage repair group. For debridement, females had a 1.2‑fold higher risk of progression than males, and older age was associated with a modest increase in risk. For microfracture, females had twice the risk of males, and age was a strong predictor of failure. Each additional year of age increased the risk of TKA by approximately 5% after debridement and 8% after microfracture. In contrast, neither age nor sex was associated with progression to TKA in the cartilage repair or unicompartmental arthroplasty subgroups. Overall, among UKB participants who underwent debridement or microfracture, women and older individuals consistently demonstrated a higher risk of conversion to TKA.

**Table 3.** Cox Proportional Hazards Regression for Progression to TKA.

Procedure	*n* (events)	Predictor	HR	95% CI	*P* value
Cartilage repair	64 (10)	Female vs male	1.22	0.34-4.35	0.75
		Age (per year)	0.99	0.93-1.05	0.74
Debridement	3,251 (759)	Female vs male	1.21	1.04-1.32	0.011
		Age (per year)	1.05	1.04-1.06	<0.001
Microfracture	382 (57)	Female vs male	2.17	1.23-3.70	0.007
		Age (per year)	1.08	1.05-1.12	<0.001
Unicompartmental	409 (6)	Female vs male	1.00	0.20-4.95	0.997
		Age (per year)	0.91	0.81-1.01	0.077

### Effect of Sex and Age on Survival After Microfracture Versus Debridement

A Cox proportional hazards model indicated that sex, age, and procedure type were significant predictors of time to TKA. Females had a higher hazard compared with males (HR = 1.19, 95% CI: 1.03-1.38, *P* = 0.021). Microfracture was associated with a significantly lower hazard compared with debridement (HR = 0.51, 95% CI: 0.31-0.83, *P* = 0.0063). Age remained strongly associated with risk, with each additional year increasing the hazard by approximately 5% (HR = 1.05, 95% CI: 1.04-1.06, *P* < 0.0001). The interaction between sex and procedure suggested that the effect of sex was larger for microfracture than for debridement (relative HR = 1.72, 95% CI: 0.95-3.11, *P* = 0.0753).

## Discussion

In this large UKB cohort, we identified substantial heterogeneity in surgical pathways for knee cartilage procedures, with nearly one in 10 participants undergoing multiple operations. Across the four surgical subgroups, clear sex‑specific differences emerged. Women who underwent debridement or microfracture had significantly higher long‑term risk of conversion to TKA, findings supported by both Kaplan-Meier estimates and Cox regression. Older age at surgery was also associated with increased risk within these procedure groups. In contrast, no clear sex‑ or age‑related associations with progression to TKA were observed for participants undergoing cartilage repair procedures or unicompartmental arthroplasty, likely reflecting smaller sample sizes and fewer outcome events. Overall, these findings indicate that progression to TKA varies by procedure type and that sex‑ and age‑related associations are most apparent following debridement and microfracture.

Our findings underscore the importance of sex-stratified analyses when evaluating long-term outcomes following articular cartilage-related procedures. The higher risk of progression to TKA observed among women may reflect a combination of anatomical, physiological, and behavioural factors. Structural differences such as narrower tibial plateaus, smaller patellae, and a shallower intercondylar notch have been proposed to alter knee joint biomechanics and may increase susceptibility to degeneration following cartilage injury.^24,33^ Hormonal influences, differences in pain perception, and variations in activity levels may further contribute to these disparities.^20,34^ Evidence from previous studies supports this trend, with men generally achieving better symptom relief and functional improvement following microfracture compared with women.^35^ Cultural expectations around knee function could contribute to differences in how men and women experience or report their symptoms. However, as UKB does not include data on functional expectations, occupational demands or activity modification at the time of surgery, these factors could not be evaluated directly. Consequently, our findings describe observed postoperative outcomes rather than the mechanisms underpinning sex‑specific differences.

Age at surgery also emerged as an important factor associated with progression to TKA, particularly among participants undergoing debridement. While age is commonly considered when counselling patients regarding surgical outcomes, previous work suggests that chronological age alone is not necessarily a marker of poor postoperative recovery.^36^ In arthroplasty populations, preoperative physical function and comorbidity burden may be more influential than age per se, although these findings may not directly translate to cartilage procedure populations. Within UKB, older individuals were more likely to progress to TKA following debridement, and women consistently showed poorer joint survival than men. Microfracture was associated with a lower hazard of TKA compared with debridement, suggesting that procedure selection may influence long‑term outcomes in appropriately selected patients.

In our study, the interaction between sex and procedure type suggested a potential trend towards a larger risk difference between females and males when undergoing microfracture compared with the difference between them when undergoing debridement, although our findings were not statistically significant. This observation should therefore be interpreted cautiously but warrants further investigation to determine whether sex‑specific biological or biomechanical factors modify the effectiveness of microfracture techniques.

The observed heterogeneity in surgical pathways indicates that there is no uniform strategy, and some patients undergo multiple procedures before TKA. Women and older participants might be over-represented in complex, multi-step pathways, reflecting initial attempts at joint preservation that fail. This variability reinforces the central finding of the present study: sex and age are associated with differential long‑term risk of TKA following certain cartilage procedures, and differences in care pathways may contribute to the observed survival patterns.

In interpreting our sex‑specific differences finding, it is also important to consider the broader clinical decision‑making context. Progression to TKA is influenced not only by symptom severity and structural disease but also by patient age, anticipated implant longevity, and shared decision‑making between clinicians and patients. Previous studies indicate that women, particularly at younger ages, report higher postoperative pain levels and lower satisfaction after TKA compared with men, even when functional outcomes are similar.^37-39^ Such findings may influence shared decision‑making and patient counselling rather than constituting a contraindication to surgery. Nevertheless, these considerations may delay or reduce the likelihood of TKA being offered, independent of underlying disease severity. Such considerations should be taken into account when interpreting associations observed in this study.

There are limitations to our study. The UKB is an invaluable resource for large-scale health research, tracking over 500,000 middle-aged participants to explore how genetic, lifestyle, and health factors influence disease over time.^25,26^ However, UKB participants are predominantly white British and under-represent younger individuals. This relatively narrow cohort means our findings may not fully reflect surgical outcomes in younger age groups. Residual confounding must also be considered when interpreting these findings. Although UKB provides extensive demographic and health‑related information, several factors that are highly relevant to both the choice of cartilage procedure and the subsequent likelihood of TKA were not available. These include pre‑operative symptom severity, functional status, cartilage defect size, lesion location, meniscal status, and joint alignment, as well as surgeon‑specific decision‑making criteria. Such factors may influence both exposure and outcome, and their absence from the models means that unmeasured differences between patient groups could partly account for the associations observed. Despite these drawbacks, the UKB dataset remains the foundation for population health research.

Female sex and older age at surgery were associated with a higher risk of progression to TKA following knee cartilage surgery in this large UKB cohort. Sex‑specific differences in time to TKA were most evident among participants undergoing debridement and microfracture, whereas no clear associations were observed for cartilage repair procedures or unicompartmental arthroplasty. These findings describe associations observed within the constraints of available UKB data and highlight the importance of considering sex, age, and procedure type when evaluating long‑term joint preservation following cartilage surgery. Further research is needed to clarify the mechanisms underlying these associations and to determine whether tailored surgical or postoperative strategies can improve long‑term outcomes.

## Supplemental Material

sj-docx-1-car-10.1177_19476035261450100 – Supplemental material for Sex and Age Affect Progression to Total Knee Arthroplasty After Cartilage Surgery: A UK Biobank Cohort Study Supplemental material, sj-docx-1-car-10.1177_19476035261450100 for Sex and Age Affect Progression to Total Knee Arthroplasty After Cartilage Surgery: A UK Biobank Cohort Study by Nicola J. Kuiper, Charlotte H. Hulme, Martyn Snow, Martin Frisher, Karina T. Wright and Jan Herman Kuiper in CARTILAGE

sj-docx-2-car-10.1177_19476035261450100 – Supplemental material for Sex and Age Affect Progression to Total Knee Arthroplasty After Cartilage Surgery: A UK Biobank Cohort Study Supplemental material, sj-docx-2-car-10.1177_19476035261450100 for Sex and Age Affect Progression to Total Knee Arthroplasty After Cartilage Surgery: A UK Biobank Cohort Study by Nicola J. Kuiper, Charlotte H. Hulme, Martyn Snow, Martin Frisher, Karina T. Wright and Jan Herman Kuiper in CARTILAGE
