# Supplementary material for: Sex and Age Affect Progression to Total Knee Arthroplasty After Cartilage Surgery: A UK Biobank Cohort Study
Source: Cartilage. 2026 Jun 29:19476035261450100. Online ahead of print. doi: 10.1177/19476035261450100 (PMC13314653; doi:10.1177/19476035261450100)
Supplement: sj-docx-1-car-10.1177_19476035261450100 – Supplemental material for Sex and Age Affect Progression to Total Knee Arthroplasty After Cartilage Surgery: A UK Biobank Cohort Study [file sj-docx-1-car-10.1177_19476035261450100.docx]

**Appendix 2. Criteria used for data extraction for participants undergoing knee surgery**

|  | Office of Population Censuses and Surveys (OPCS) Classification of Surgical Operations and Procedures, 4th revision |
| --- | --- |
| Knee joint | Z846 |
| Laterality | Z942, Z943 |
| Anatomical compartment of knee joint | Z765, Z766, Z844, Z845 |
| Autologous Chondrocyte Implantation (ACI) | W714, W892, Y703, Y363, Y365, Y367, Y711 |
| Autologous Matrix-Induced Chondrogenesis (AMIC) | O191, W718, Y667 |
| Mosaicplasty | W837 |
| Debridement | W802, W833, W836, W718, Y055, Y677 |
| Chondroplasty | W833, W834, W835, W836, W891 |
| Microfracture | W711, W845 |
| Unicompartmental joint replacement | W581 |
| Total knee arthroplasty (primary) | O181, W401, W408, W409, W411, W418, W419, W42.1, W428, W429 |
